# Exponential stability for neutral stochastic functional partial differential equations driven by Brownian motion and fractional Brownian motion

**DOI:** 10.1186/s13660-018-1793-9

**Published:** 2018-08-01

**Authors:** Xinwen Zhang, Dehao Ruan

**Affiliations:** 1 Department of Mathematics, Guangzhou College of Commerce, Guangzhou, P.R. China; 2 Department of Probability and Statistics, School of Mathematics and Information Science, Guangzhou University, Guangzhou, P.R. China

**Keywords:** 34k40, 35B35, 47D03, 60H15, Neutral stochastic functional partial differential equation, Mild solution, Exponential stability, Brownian motion, Fractional Brownian motion

## Abstract

In this paper, we study the exponential stability in the *p*th moment of mild solutions to neutral stochastic functional partial differential equations driven by Brownian motion and fractional Brownian motion:

$$ d \bigl[x(t)+g(t,x_{t}) \bigr]= \bigl[Ax(t)+f(t,x_{t}) \bigr] \,dt+h(t,x_{t})\,dW(t)+\sigma(t)\,dB^{H}(t), $$

where $H\in(1/2,1)$. Our method for investigating the stability of solutions is based on the Banach fixed point theorem. The obtained results generalize and improve the results due to Boufoussi and Hajji (Stat. Probab. Lett. 82:1549–1558, 2012), Caraballo et al. (Nonlinear Anal. 74:3671–3684, 2011), and Luo (J. Math. Anal. Appl. 355:414–425, 2009).

## Introduction

Many dynamical systems not only depend on present and past states but also involve derivatives with delays. Neutral stochastic functional partial differential equations (NSFPDEs) are often used to describe such kind of systems. In recent years, NSFPDEs have been extensively studied in the literature, we can refer to [6, 9, 12–14, 19] for those only driven by Brownian motion and also refer to [1, 2, 4, 5, 11] for those only driven by fractional Brownian motion (fBm). For example, Luo [13] studied the exponential stability in mean square of mild solution for NSFPDE only driven by Brownian motion; Boufoussi and Hajji [2] discussed the exponential stability in mean square of mild solution for NSPDE only driven by fBm with finite delay. Furthermore, the stochastic processes in hydrodynamics, telecommunications, and finance demonstrate the availability of random noise that can be modeled by Brownian motion and also the so-called long memory that can be modeled with the help of fBm with Hurst index $1/2< H<1$. Since the seminal paper [7], mixed stochastic models containing both standard Brownian motion and fBm have gained a lot of attention. Very recently, there has been considerable interest in studying this class of SDEs (see [3, 10, 16, 17, 20, 21]).

However, to the best of our knowledge, there is no paper which investigates the exponential stability in the *p*th moment of mild solutions to neutral stochastic functional partial differential equations driven by Brownian motion and fractional Brownian motion. Motivated by the above, in this work, we consider the following mixed NSFPDE:

1.1 $$ \textstyle\begin{cases} d[x(t)+g(t,x_{t})]=[Ax(t)+f(t,x_{t})]\,dt+h(t,x_{t})\,dW(t)+\sigma(t)\, dB^{H}(t),\\ \quad t\in[0,T],\\ x(s)=\varphi(s), \quad s\in[-r,0], r\geq0, \end{cases} $$

under suitable conditions on the operator *A*, the coefficient functions *g*, *f*, *h*, *σ*, and the initial value *φ*. Here $W(t)$ denotes Brownian motion and $B^{H}(t)$ denotes fBm with $H\in(1/2,1)$.

The purpose of this paper is to investigate the exponential stability in the *p*th moment of mild solution of mixed NSFPDE (1.1) by means of the Banach fixed point theory.

The rest of this paper is organized as follows. In Sect. 2, we first recall some necessary preliminaries on the stochastic differential equations with respect to Brownian motion and fractional Brownian motion. In Sect. 3, the exponential stability in the *p*th moment of mild solution of mixed NSFPDE (1.1) is proved, the results in [2, 5, 13] are generalized and improved.

## Preliminaries

Let $T>0$ be a fixed time horizon and $(\Omega,\mathcal{F}, \mathbb {P})$ be a complete probability space equipped with a normal filtration $\mathcal{F}=\{\mathcal{F}_{t}\}_{t\geq0}$ satisfying the usual assumptions. Let $W=\{W(t),t\in[0,T]\}$ be a standard Brownian motion and $B=\{B^{H}(t),t\in[0,T]\}$ be a fractional Brownian motion with Hurst parameter $H\in(1/2,1)$ on the complete probability space $(\Omega ,\mathcal{F},\mathbb{P})$. We denote by $C([-r,T]; U)$ the space of all continuous functions from $[-r,T]$ to *U*. Let $(U,\|\cdot\|_{U},(\cdot,\cdot)_{U})$ and $(K_{i},\|\cdot\|_{K_{i}},(\cdot,\cdot)_{K_{i}})$ be two separable Hilbert spaces, and let $L(K_{i},U)$ denote the space of all bounded linear operators from $K_{i}$ to *U*, $i=W,B$. We assume that $\{e_{n}^{(i)}\} _{n\in\mathbb{N^{+}}}$ are two complete orthonormal bases in $K_{i}$ and $Q^{(i)}\in L_{i}^{0}(K_{i},U)$ are two operators defined by $Q^{(i)}e_{n}^{(i)}=\lambda_{n}^{(i)}e_{n}^{(i)}$ with finite trace $\operatorname{tr}Q^{(i)}=\sum_{n=1}^{\infty}\lambda_{n}^{(i)}<\infty$, where $\{ \lambda_{n}^{(i)}\}_{n\in\mathbb{N^{+}}}$ are non-negative real numbers and $i=W,B$. Then there exists a real-valued sequence $\{\omega_{n}(t)\}_{n\in \mathbb{N^{+}}}$ of one-dimensional standard Brownian motions mutually independent over $(\Omega,\mathcal{F},\mathbb{P})$ such that

$$W(t)=\sum_{n=1}^{\infty}\sqrt{ \lambda_{n}^{(W)}}e_{n}^{(W)}\omega _{n}(t),\quad t\geq0. $$

The infinite dimensional cylindrical $K_{B}$-valued fBm $B^{H}(t)$ is defined by the formal sum

$$B^{H}(t)=\sum_{n=1}^{\infty}\sqrt { \lambda_{n}^{(B)}}e_{n}^{(B)}w_{n}^{H}(t),\quad t\geq0, $$

where the sequence $\{w_{n}^{H}(t)\}_{n\in\mathbb{N^{+}}}$ are stochastically independent scalar fBms with Hurst parameter $H\in(1/2,1)$. Let $L_{i}^{0}(K_{i},U)$ be the space of all $Q^{(i)}$-Hilbert–Schmidt operators from $K_{i}$ to *U*, $i=W,B$. Now we can show the following two definitions of norms.

### Definition 2.1

(Chen et al. [6])

Let $\xi\in L(K_{W},U)$ and define

$$\Vert \xi \Vert _{ L_{W}^{0}}^{2}:=\operatorname{tr} \bigl(\xi Q^{(W)}\xi * \bigr)=\sum_{n=1}^{\infty}\Bigl\Vert \sqrt{\lambda_{n}^{(W)}}\xi e_{n}^{(W)} \Bigr\Vert _{U}^{2}. $$

If $\|\xi\|_{ L_{W}^{0}}^{2}<\infty$, then *ξ* is called a $Q^{(W)}$-Hilbert–Schmidt operator and the space $L_{W}^{0}:= L_{W}^{0}(K_{W},U)$ equipped with the inner product $\langle\varphi,\psi \rangle_{ L_{W}^{0}}=\sum_{n=1}^{\infty}\langle\varphi e_{n}^{(W)},\psi e_{n}^{(W)}\rangle$ is a separable Hilbert space.

### Definition 2.2

(Boufoussi and Hajji [2])

In order to define Wiener integrals with respect to the $Q^{(B)}$-fBm, we recall that $\eta\in L(K_{B},U)$ is called a $Q^{(B)}$-Hilbert–Schmidt operator if

$$\Vert \eta \Vert _{ L_{B}^{0}}^{2}:=\operatorname{tr} \bigl(\eta Q^{(B)}\eta * \bigr)=\sum_{n=1}^{\infty}\Bigl\Vert \sqrt{\lambda_{n}^{(B)}}\eta e_{n}^{(B)} \Bigr\Vert _{U}^{2}< \infty, $$

and that the space $L_{B}^{0}:= L_{B}^{0}(K_{B},U)$ equipped with the inner product $\langle\varphi,\psi\rangle_{ L_{B}^{0}}=\sum_{n=1}^{\infty}\langle\varphi e_{n}^{(B)}, \psi e_{n}^{(B)}\rangle$ is a separable Hilbert space.

### Lemma 2.1

(Prato and Zabczyk [8])

*For any* $p\geq0$ *and for arbitrary* $L_{W}^{0}$-*valued predictable process* $\Phi(\cdot)$, *we have*

$$\sup_{s\in[0,t]}E \biggl\Vert \int_{0}^{s}\Phi(u)\,dW(u) \biggr\Vert _{U}^{2p}\leq c_{p} \biggl( \int_{0}^{t} \bigl(E \bigl\Vert \Phi(s) \bigr\Vert _{L_{W}^{0}}^{2p} \bigr)^{1/p}\,ds \biggr)^{p}, \quad t\in[0,T], $$

*where* $c_{p}=(p(2p-1))^{p}$.

Let $\{w^{H}(t)\}_{t\in[0,T]}$ be the one-dimensional fBm with Hurst parameter $H\in(1/2,1)$. This means by definition that $w^{H}$ is a centered Gaussian process with covariance function:

$$ R_{H}(s,t)=\frac{1}{2} \bigl(t^{2H}+s^{2H}- \vert t-s \vert ^{2H} \bigr). $$

Moreover, $w^{H}$ has the following Wiener integral representation:

$$ w^{H}(t)= \int_{0}^{t}K_{H}(t,s)\,dw(s), $$

where $w=\{w(t)\}_{t\in[0,T]}$ is a Wiener process and $K_{H}(t,s)$ is the kernel given by

$$K_{H}(t,s)=c_{H}s^{\frac{1}{2}-H} \int_{s}^{t}(u-s)^{H-\frac{3}{2}}u^{H-\frac {1}{2}}\,du $$

for $t>s$. Here, $c_{H}=\sqrt{\frac{H(2H-1)}{\mathcal{B}(2-2H,H-\frac {1}{2})}}$ and $\mathcal{B}(\cdot,\cdot)$ denotes the beta function. We put $K_{H}(t,s)=0$ if $t\leq s$.

### Lemma 2.2

(Caraballo et al. [5])

*Let* $\varphi: [0,T]\longmapsto L^{0}_{B}(K_{B},U)$ *such that*

2.1 $$ \sum^{\infty}_{n=1} \bigl\Vert \bigl(\varphi \sqrt{Q^{(B)}}e_{n} \bigr) \bigr\Vert _{L^{1/H}([0,T];U)}< \infty $$

*holds*, *and for any* *a*, $b\in[0,T]$ *with* $a>b$,

$$\mathbb{E} \biggl\Vert \int^{a}_{b}\varphi(s)\,dB^{H}(s) \biggr\Vert ^{2}_{U}\leq c{_{H}}H(2H-1) (a-b)^{2H-1}\sum^{\infty}_{n=1} \int^{a}_{b} \bigl\Vert \varphi(s)\sqrt {Q^{(B)}}e_{n} \bigr\Vert ^{2}_{U} \,ds, $$

*where* $c_{H}=\sqrt{\frac{H(2H-1)}{\mathcal{B}(2-2H,H-\frac{1}{2})}}$. *If*, *in addition*,

$$ \sum^{\infty}_{n=1} \bigl\Vert \varphi(t) \sqrt {Q^{(B)}}e_{n} \bigr\Vert _{U} \textit{ is uniformly convergent for } t\in[0,T], $$

*then*

2.2 $$ \mathbb{E} \biggl\Vert \int^{a}_{b}\varphi(s)\,dB^{H}(s) \biggr\Vert ^{2}_{U}\leq c_{H}H(2H-1) (a-b)^{2H-1} \int^{a}_{b} \bigl\Vert \varphi(s) \bigr\Vert ^{2}_{L^{0}_{B}(K,U)}\,ds. $$

### Lemma 2.3

(Mémin et al. [15])

*For every* *T*, $\int^{T}_{0}f(t)\,dZ_{t}$ *is a centered Gaussian random variable*, *for every* $p>0$, *there exists a constant* $k(p)$ *such that*

2.3 $$ \mathbb{E} \biggl\Vert \int^{T}_{0}f(t)\,dZ_{t} \biggr\Vert ^{p}\leq k(p) \biggl(\mathbb{E} \biggl\Vert \int^{T}_{0}f(t)\,dZ_{t} \biggr\Vert ^{2} \biggr)^{\frac{p}{2}}. $$

### Lemma 2.4

(Pazy [18])

*Suppose that* *A* *is the infinitesimal generator of an analytic semigroup of uniformly bounded linear operators* $\{S(t)\}_{t\geq0}$ *on the separable Hilbert space* *U*. *It is well known that there exist some constants* $M\geq1,\lambda\in\mathbb {R}$ *such that* $\|S(t)\|\leq Me^{\lambda t}$, *for* $t\geq0$, *and moreover*, *if* $0\in\rho(-A)$, *where* $\rho(-A)$ *is the resolvent set of* −*A*, *then*,

- *for any* $c\geq0$, *the subspace* $D((-A)^{c})$ *is dense in* *U* *with the norm*
  $$\Vert \zeta \Vert _{c}^{2}:=\sup_{t\in\mathbb {R}} \mathbb {E} \bigl\Vert (-A)^{c} \zeta \bigl(t,x(t) \bigr) \bigr\Vert _{U}, \zeta\in D \bigl((-A)^{c} \bigr), $$
- *for each* $x\in D((-A)^{c})$, *we have* $S(t)(-A)^{c} x=(-A)^{c} S(t)x$,
- *there exist a pair of positive constants* $M_{c}>0$ *and* $\lambda>0$ *such that*
  $$\bigl\Vert (-A)^{c} S(t) \bigr\Vert _{U}\leq M_{c} e^{-\lambda t}t^{-c},\quad t>0. $$

We denote by $C([a,b];U)=C([a,b]; (\Omega,\mathcal{F},\mathbb {P};U))$ the Banach space of all continuous functions from $[a,b]$ into *U* endowed with the supremum norm.

Consider two fixed real numbers $r\geq0$ and $T>0$. If $x\in C([-r,T];U)$ for each $t\in[0,T]$, we denote by $x_{t}\in C([-r,0];U)$ the function defined by $x_{t}(s)=x(t+s)$ for $s\in[-r,0]$.

We consider the exponential stability of mild solution to the following mixed NSFPDE:

2.4 $$ \textstyle\begin{cases}d[x(t)+g(t,x_{t})]=[Ax(t)+f(t,x_{t})]\,dt+h(t,x_{t})\, dW(t)+\sigma(t)\,dB^{H}(t),\\ \quad t\in[0,T],\\ x(t)=\varphi(t), \quad t\in[-r,0], \end{cases} $$

where $W(t)$ is the Brownian motion and $B^{H}(t)$ is the fractional Brownian motion which were previously introduced, the initial value $\varphi\in C([-r,0];U)$, and *A*: Dom(*A*) $\subset U\rightarrow U$ is the infinitesimal generator of a strongly continuous semigroup $S(\cdot)$ on *U*. The mappings $f:[0,T]\times C([-r,0];U)\rightarrow U$, $g:[0,T]\times C([-r,0];U)\rightarrow U$, $h:[0,T]\times C([-r,0];U)\rightarrow U$, and $\sigma: [0,T]\rightarrow L_{B}^{0}(K_{B},U)$, and they are all Borel measurable.

### Definition 2.3

A U-valued process $\{x(t), t\in[-r,T]\}$ is called mild solution of (2.4) if

(i) $x(t)$ is adapted to $\mathcal{F}_{t}$, $t\geq0$;
(ii) $x(t)=\varphi(t)$ for $t\in[-r,0]$;
(iii) $x(t)\in U$ has càdlàg paths on $t\in[0,T]$ almost surely, and for arbitrary $t\in[0,T]$,
  2.5 $$\begin{aligned} x(t)={}&S(t) \bigl[\varphi(0)+g(0,\varphi) \bigr]-g(t,x_{t}) \\ &{}- \int ^{t}_{0}AS(t-s)g(s,x_{s})\,ds+ \int^{t}_{0}S(t-s)f(s,x_{s})\,ds \\ &{}+ \int^{t}_{0}S(t-s)h(s,x_{s})\,dW(s)+ \int^{t}_{0}S(t-s)\sigma(s)\,dB^{H}(s)\quad \mbox{a.s.} \end{aligned}$$

### Definition 2.4

Let *p* be an integer $p\geq2$. Equation (2.5) is said to be exponentially stable in the *p*th moment if, for any initial value *φ*, there exists a pair of constants $\gamma>0$ and $C>0$ such that

2.6 $$ \mathbb{E} \bigl\Vert x(t) \bigr\Vert _{U}^{p}\leq Ce^{-\gamma t},\quad t\geq0. $$

In order to set the stability problem, we suppose that the following assumptions hold:

(H1): The operator *A* is a closed linear operator generating a strongly continuous semigroup $S(t)$, $t\geq0$, on the separable Hilbert space *U* and satisfying
  2.7 $$ \bigl\Vert S(t) \bigr\Vert _{U}\leq Me^{-\lambda t},\quad \forall t\geq0, \text{ where } M\geq1 \text{ and } \lambda>0. $$
(H2): The mappings $f(t,\cdot)$ and $h(t,\cdot)$ satisfy the following conditions: $p\geq2$ and *p* is an integer for any $x,y\in C([-r,T];U)$ and $t\geq0$
  2.8 $$\begin{aligned} &\int^{t}_{0}e^{\lambda s} \bigl\Vert f(t,x_{s})-f(t,y_{s}) \bigr\Vert ^{p}_{U} \,ds\leq C^{p}_{f} \int^{t}_{-r}e^{\lambda s} \bigl\Vert x(s)-y(s) \bigr\Vert ^{p}_{U}\,ds, \quad C_{f}\geq0, \end{aligned}$$
  2.9 $$\begin{aligned} &\int^{t}_{0}e^{\lambda s} \bigl\Vert h(t,x_{s})-h(t,y_{s}) \bigr\Vert ^{p}_{U} \,ds\leq C^{p}_{h} \int^{t}_{-r}e^{\lambda s} \bigl\Vert x(s)-y(s) \bigr\Vert ^{p}_{U}\,ds,\quad C_{h}\geq0, \\ &\int_{0}^{\infty}e^{\lambda s} \bigl\Vert f(s,0) \bigr\Vert ^{p}_{U}\,ds< \infty. \end{aligned}$$
(H3): The mapping $g:[0,T]\times C([-r,0];U)\rightarrow U$ is continuous in the *p*th mean sense and satisfies, for any $x,y\in C([-r,T];U)$ and $t\geq0$, $g(t,x)\in D((-A)^{\beta})$ and
  2.10 $$ \begin{aligned} &\bigl\Vert (-A)^{\beta}g(t,x)-(-A)^{\beta}g(t,y) \bigr\Vert _{U}\leq C_{g} \Vert x-y \Vert _{U},\quad C_{g}\geq0, \\ &\lim_{t\rightarrow s}\mathbb {E} \bigl\Vert (-A)^{\beta}g(t,x)-(-A)^{\beta}g(s,x) \bigr\Vert _{U}^{p}=0, \end{aligned} $$
  where $\beta\in(0,1]$ and satisfies $p\beta>1$, *p* is an integer $p\geq 2$. We further assume $g(t,0)\equiv0$ for $t\geq0$.
(H4): The mapping *σ*: $[0,T]\rightarrow L^{0}_{B}(K_{B},U)$ satisfies
  2.11 $$ \int^{\infty}_{0}e^{\lambda s} \bigl\Vert \sigma(s) \bigr\Vert ^{2}_{L^{0}_{B}(K_{B},U)}\,ds< \infty. $$

## Main results

In this section, we consider the exponential stability in the *p*th moment of mild solution of mixed NSFPDE (2.4) by means of the Banach fixed point theory.

### Theorem 1

*Suppose that conditions* (H1)–(H4) *hold*. *Then Eq*. (2.4) *is exponentially stable in the* *pth moment if*

3.1 $$\begin{aligned} &4^{p-1} \biggl( \bigl\Vert (-A)^{-\beta} \bigr\Vert ^{p}_{U}C^{p}_{g}+C^{p}_{g}M^{p}_{1-\beta} \lambda^{-p\beta}\Gamma^{p-1} \biggl(\frac {p\beta-1}{p-1} \biggr) +M^{p}\lambda^{-p}C_{f}^{p} \\ & \quad{}+M^{p}c_{p}C_{h}^{p} \lambda^{-p/2} \bigl(2{(p-1)/(p-2)} \bigr)^{1-p/2} \biggr)< 1, \end{aligned}$$

*where* $\Gamma(\cdot)$ *is the gamma function and* $M,M_{1-\beta}$ *are the corresponding constants in Lemma *2.4, *and* $c_{p}=(p(p-1)/2)^{p/2}$.

### Proof

Denote by $\mathcal{S}$ the Banach space of all $\mathcal{F}$-adapted processes $\phi(t,w):[-r,\infty)\times\Omega \longrightarrow\mathbb{R}$, which is almost surely continuous in *t* for fixed $\omega\in\Omega$. Moreover, $\phi (s,w)=\varphi(s)$ for $s\in[-r,0]$ and $e^{\alpha t}\mathbb{E}\|\phi (t,w)\|^{p}_{U}\longrightarrow0$ as $t\longrightarrow\infty$, where *α* is a positive constant such that $0<\alpha<\lambda$.

Define an operator $\pi: \mathcal{S}\longrightarrow\mathcal{S}$ by $(\pi x)(t)=\psi(t)$ for $t\in[-r,0]$ and for $t\geq0$,

3.2 $$\begin{aligned} (\pi x) (t) ={}&S(t) \bigl[\varphi(0)+g(0,\varphi) \bigr]-g(t,x_{t})- \int ^{t}_{0}AS(t-s)g(s,x_{s})\,ds \\ &{}+ \int^{t}_{0}S(t-s)f(s,x_{s})\,ds+ \int^{t}_{0}S(t-s)h(s,x_{s})\,dW(s)+ \int ^{t}_{0}S(t-s)\sigma(s)\,dB^{H}(s) \\ :={}&\sum^{6}_{i=1}I_{i}(t). \end{aligned}$$

Firstly, we verify the continuity in the *p*th moment of *π* on $[0,\infty)$. Let $x\in\mathcal{S}$, $t_{1}\geq0$, and r be positive and small enough, then

$$\mathbb{E} \bigl\Vert (\pi x) (t_{1}+r)-(\pi x) (t_{1}) \bigr\Vert ^{p}_{U}\leq6^{p-1}\mathbb{e}\sum ^{6}_{i=1}\mathbb{E} \bigl\Vert I_{i}(t_{1}+r)-I_{i}(t_{1}) \bigr\Vert ^{p}_{U}. $$

Obviously,

$$ \mathbb{E} \bigl\Vert I_{i}(t_{1}+r)-I_{i}(t_{1}) \bigr\Vert ^{p}_{U}\longrightarrow 0,\quad i=1, 4, \text{ as } r\longrightarrow0. $$

Since the operator $(-A)^{-\beta}$ is bounded and by (H3) we know the mapping $(-A)^{\beta}g$ is continuous in the *p*th moment, so

$$ \mathbb{E} \bigl\Vert I_{2}(t_{1}+r)-I_{2}(t_{1}) \bigr\Vert ^{p}_{U}\longrightarrow 0,\quad \text{as } r \longrightarrow0. $$

As for the third term on the right-hand side of (3.2), we get

$$\begin{aligned} \mathbb{E} \bigl\Vert I_{3}(t_{1}+r)-I_{3}(t_{1}) \bigr\Vert ^{p}_{U} \leq{}& 2^{p-1}\mathbb{E} \biggl\Vert \int^{t_{1}}_{0} \bigl(S(r)-I \bigr) (-A)^{1-\beta }S(t_{1}-s) (-A)^{\beta}g(s,x_{s}) \,ds \biggr\Vert _{U}^{p} \\ & {}+2^{p-1}\mathbb{E} \biggl\Vert \int^{t_{1}+r}_{t_{1}}(-A)^{1-\beta }S(t_{1}+r-s) (-A)^{\beta}g(s,x_{s})\,ds \biggr\Vert _{U}^{p} \\ :={}&I_{31}(r)+I_{32}(r). \end{aligned}$$

By the strong continuity of $S(t)$, for any $s\in[0,t_{1}]$, we have

$$ \lim_{r\rightarrow0} \bigl(S(r)-I \bigr) (-A)^{1-\beta }S(t_{1}-s) (-A)^{\beta}g(s,x_{s})=0. $$

By using Lemma 2.4 and the fact that $0<\beta\leq1$, we have

$$ \bigl\Vert \bigl(S(r)-I \bigr) (-A)^{1-\beta}S(t_{1}-s) (-A)^{\beta}g(s,x_{s}) \bigr\Vert _{U}\leq \frac{2MM_{1-\beta}}{(t_{1}-s)^{1-\beta}} \bigl\Vert (-A)^{\beta}g(s,x_{s}) \bigr\Vert _{U}, $$

since $\beta\in(0,1]$ and by the Lebesgue dominated theorem, we obtain

$$ \lim_{r\rightarrow0}I_{31}(r)=0. $$

On the other hand,

$$ \bigl\Vert (-A)^{1-\beta}S(t_{1}+r-s) (-A)^{\beta}g(s,x_{s}) \bigr\Vert _{U}\leq \frac{M_{1-\beta}}{(t_{1}+r-s)^{1-\beta}} \bigl\Vert (-A)^{\beta}g(s,x_{s}) \bigr\Vert _{U}, $$

so $I_{32}(r)\longrightarrow0$ as $r\longrightarrow0$, then

$$ \mathbb{E} \bigl\Vert I_{3}(t_{1}+r)-I_{3}(t) \bigr\Vert ^{p}_{U}\longrightarrow 0,\quad \text{as } r \longrightarrow0. $$

Moreover, by using Lemma 2.1, we get

$$\begin{aligned} & E \bigl\Vert I_{5}(t_{1}+r)-I_{5}(t_{1}) \bigr\Vert _{U}^{p} \\ &\quad =E \biggl\Vert \int_{0}^{t_{1}} \bigl(S(t_{1}+r-s)-S(t_{1}-s) \bigr)g(s,x_{s})\,dW(s) \\ &\qquad{}+ \int_{t_{1}}^{t_{1}+r}S(t_{1}+r-s)g(s,x_{s}) \,dW(s) \biggr\Vert _{U}^{p} \\ &\quad \leq2^{p-1}c_{p} \biggl[ \int_{0}^{t_{1}} \bigl(E \bigl\Vert \bigl(S(t_{1}+r-s)-S(t_{1}-s) \bigr)g(s,x_{s} ) \bigr\Vert _{U}^{p} \bigr)^{2/p}\,ds \biggr]^{p/2} \\ &\qquad{}+2^{p-1}c_{p} \biggl[ \int_{t_{1}}^{t_{1}+r} \bigl(E \bigl\Vert S(t_{1}+r-s)g(s,x_{s}) \bigr\Vert _{U}^{p} \bigr)^{2/p}\,ds \biggr]^{p/2}\to0\quad \mbox{as }r\to0, \end{aligned}$$

where $c_{p}=(p(p-1)/2)^{p/2}$.

As for the sixth term on the right-hand side of (3.2), we first verify $\mathbb{E}\|I_{6}(t_{1}+r)-I_{6}(t_{1})\|^{2}_{U}\longrightarrow0$ as $r\longrightarrow0$. Further, by using (2.3), we can get $\mathbb{E}\| I_{6}(t_{1}+r)-I_{6}(t_{1})\|^{p}_{U}\longrightarrow0$ as $r\longrightarrow0$.

By using the Cauchy–Schwarz inequality, we get

$$\begin{aligned} \mathbb{E} \bigl\Vert I_{6}(t_{1}+r)-I_{6}(t_{1}) \bigr\Vert ^{2}_{U} \leq{}&2 \mathbb{E} \biggl\Vert \int^{t_{1}}_{0} \bigl(S(t_{1}+r-s)-S(t_{1}-s) \bigr)\sigma(s)\, dB^{H}(s) \biggr\Vert ^{2}_{U} \\ &{}+2\mathbb{E} \biggl\Vert \int^{t_{1}+r}_{t_{1}}S(t_{1}+r-s)\sigma(s)\, dB^{H}(s) \biggr\Vert ^{2}_{U} \\ :={}&I_{61}(r)+I_{62}(r) . \end{aligned}$$

Applying inequality (2.2) and condition (2.7) to $J_{1}$, we get

$$\begin{aligned} I_{61}(r)& =2\mathbb{E} \biggl\Vert \int ^{t_{1}}_{0} \bigl(S(t_{1}+r-s)-S(t_{1}-s) \bigr)\sigma(s)\,dB^{H}(s) \biggr\Vert ^{2}_{U} \\ & \leq2c_{H}H(2H-1)t_{1}^{2H-1} \int^{t_{1}}_{0} \bigl\Vert S(t_{1}-s) \bigl(S(r)-I \bigr)\sigma(s) \bigr\Vert ^{2}_{L^{0}_{B}(K_{B},U)}\,ds \\ & \leq2c_{H}H(2H-1)t_{1}^{2H-1}M^{2} \int^{t_{1}}_{0} \bigl\Vert \bigl(S(r)-I \bigr) \sigma(s) \bigr\Vert ^{2}_{L^{0}_{B}(K_{B},U)}\,ds\longrightarrow0, \end{aligned}$$

when $r\longrightarrow0$ since $S(r)\sigma(s)\longrightarrow\sigma (s)$ and $\|S(r)\sigma(s)\|_{L^{0}_{B}(K_{B},U)}\leq M\|\sigma(s)\| _{L^{0}_{B}(K_{B},U)}$ for any fixed $s>0$.

Applying inequality (2.2) and condition (2.7) to $J_{2}$, we can obtain

$$I_{62}(r)\leq2c_{H}H(2H-1)r^{2H-1}M^{2} \int^{t_{1}+r}_{t_{1}}\bigl\| \sigma(s)\bigr\| ^{2}_{L^{0}_{B}(K_{B},U)} \,ds\longrightarrow0 \quad \mbox{as } r\longrightarrow0. $$

So, $\mathbb{E}\|I_{6}(t_{1}+r)-I_{6}(t_{1})\|^{2}_{U}\longrightarrow0$ as $r\longrightarrow0$.

Further, by using (2.3), we get

$$ \mathbb{E} \bigl\Vert I_{6}(t_{1}+r)-I_{6}(t_{1}) \bigr\Vert ^{p}_{U}\leq k(p) \bigl[\mathbb{E} \bigl\Vert I_{6}(t_{1}+r)-I_{6}(t_{1}) \bigr\Vert ^{2}_{U} \bigr]^{\frac {p}{2}}\longrightarrow0 \quad \text{as }r\longrightarrow0. $$

Thus, *π* is indeed continuous in the *p*th moment on $[0,\infty)$.

Secondly, we show that $\pi(\mathcal{S})\subset\mathcal{S}$. It follows from (3.2) that

3.3 $$\begin{aligned} e^{\alpha t}\mathbb{E} \bigl\Vert (\pi x) (t) \bigr\Vert ^{p}_{U} \leq {}&6^{p-1}e^{\alpha t}\mathbb{E} \bigl\Vert S(t) \bigl(\varphi(0)+g(0,\varphi) \bigr) \bigr\Vert ^{p}_{U} \\ & {}+6^{p-1}e^{\alpha t}\mathbb{E} \bigl\Vert g(t,x_{t}) \bigr\Vert ^{p}_{U} \\ & {}+6^{p-1}e^{\alpha t}\mathbb{E} \biggl\Vert \int^{t}_{0}AS(t-s)g(s,x_{s})\, ds \biggr\Vert ^{p}_{U} \\ & {}+6^{p-1}e^{\alpha t}\mathbb{E} \biggl\Vert \int^{t}_{0}S(t-s)f(s,x_{s})\, ds \biggr\Vert ^{p}_{U} \\ & {}+6^{p-1}e^{\alpha t}\mathbb{E} \biggl\Vert \int^{t}_{0}S(t-s)h(s,x_{s})\, dW(s) \biggr\Vert ^{p}_{U} \\ & {}+6^{p-1}e^{\alpha t}\mathbb{E} \biggl\Vert \int^{t}_{0}S(t-s)\sigma(s)\, dB^{H}(s) \biggr\Vert ^{p}_{U}. \end{aligned}$$

Now we estimate the terms on the right-hand side of (3.3). First, by condition (2.7), we can obtain

3.4 $$\begin{aligned} &6^{p-1}e^{\alpha t}\mathbb{E}\bigl\| S(t) (\varphi(0)+g(0,\varphi) \bigr\| ^{p}_{U} \\ &\quad \leq 6^{p-1}M^{p}e^{-p\lambda t}e^{\alpha t} \bigl\Vert \varphi(0)+g(0,\varphi) \bigr\Vert _{U}^{p} \rightarrow0\quad \text{as } t\rightarrow\infty. \end{aligned}$$

For any $x(t)\in\mathcal{S}$ and any $\varepsilon_{1}>0$, there exists $t_{1}>0$ such that $e^{\alpha t}\mathbb{E}\|x(t)\|^{p}_{U}<\varepsilon_{1}$ for $t-r>t_{1}$. Thus we can get

$$\begin{aligned} 6^{p-1}e^{\alpha t}\mathbb{E} \bigl\vert g(t,x_{t}) \bigr\vert ^{p}_{U} &\leq 6^{p-1} \bigl\Vert (-A)^{-\beta} \bigr\Vert ^{p}_{U}C^{p}_{g}e^{\alpha t} \mathbb{E} \Vert x_{t} \Vert ^{p}_{U} \\ & \leq6^{p-1} \bigl\Vert (-A)^{-\beta} \bigr\Vert ^{p}_{U}C^{p}_{g}e^{\alpha t} \mathbb{E}\sup_{-r\leq s\leq0} \bigl\Vert x(t+s) \bigr\Vert ^{p}_{U} \\ & \leq6^{p-1} \bigl\Vert (-A)^{-\beta} \bigr\Vert ^{p}_{U}C^{p}_{g}e^{\alpha t}e^{-\alpha (t+s)} \varepsilon_{1} \\ & \leq6^{p-1} \bigl\Vert (-A)^{-\beta} \bigr\Vert ^{p}_{U}C^{p}_{g}e^{\alpha s} \varepsilon_{1}. \end{aligned}$$

So, from the above, we can get

3.5 $$\begin{aligned} 6^{p-1}e^{\alpha t}\mathbb {E} \bigl\vert g(t,x_{t}) \bigr\vert ^{p}_{U} \longrightarrow0 \quad \mbox{as } t \longrightarrow\infty. \end{aligned}$$

Further, Hölder’s inequality, Lemma 2.4, and (2.10) yield

$$\begin{aligned} &6^{p-1} e^{\alpha t}\mathbb{E} \biggl\Vert \int ^{t}_{0}AS(t-s)g(s,x_{s})\,ds \biggr\Vert ^{p}_{U} \\ &\quad \leq6^{p-1}e^{\alpha t}\mathbb{E} \biggl( \int^{t}_{0} \bigl\Vert (-A)^{1-\beta }S(t-s) (-A)^{\beta}g(s,x_{s}) \bigr\Vert _{U}\,ds \biggr)^{p} \\ &\quad \leq6^{p-1}C^{p}_{g}M^{p}_{1-\beta}e^{\alpha t} \mathbb{E} \biggl[ \int ^{t}_{0}e^{-\lambda(t-s)}(t-s)^{\beta-1} \Vert x_{s} \Vert _{U}\,ds \biggr]^{p} \\ &\quad =6^{p-1}C^{p}_{g}M^{p}_{1-\beta}e^{\alpha t} \mathbb{E} \biggl[ \int ^{t}_{0}e^{-\lambda(p-1)(t-s)/p}(t-s)^{\beta-1}e^{-\lambda(t-s)/p} \Vert x_{s} \Vert _{U}\,ds \biggr]^{p} \\ &\quad \leq6^{p-1}C^{p}_{g}M^{p}_{1-\beta}e^{\alpha t} \biggl[ \int^{t}_{0}e^{-\lambda (t-s)}(t-s)^{\frac{(\beta-1)p}{p-1}}\,ds \biggr]^{p-1} \int^{t}_{0}e^{-\lambda (t-s)}\mathbb{E} \Vert x_{s} \Vert ^{p}_{U}\,ds \\ &\quad \leq6^{p-1}C^{p}_{g}M^{p}_{1-\beta} \lambda^{1-p\beta}\Gamma^{p-1} \biggl(\frac {p\beta-1}{p-1} \biggr)e^{\alpha t} \int^{t}_{0}e^{-\lambda(t-s)}\mathbb{E} \Vert x_{s} \Vert ^{p}_{U}\,ds \\ &\quad =6^{p-1}C^{p}_{g}M^{p}_{1-\beta} \lambda^{1-p\beta}\Gamma^{p-1} \biggl(\frac {p\beta-1}{p-1} \biggr)e^{\alpha t} \int^{t}_{0}e^{-\lambda(t-s)}e^{\alpha s-\alpha s} \mathbb{E} \Vert x_{s} \Vert ^{p}_{U}\,ds \\ &\quad =6^{p-1}C^{p}_{g}M^{p}_{1-\beta} \lambda^{1-p\beta}\Gamma^{p-1} \biggl(\frac {p\beta-1}{p-1} \biggr)e^{(\alpha-\lambda)t} \int^{t}_{0}e^{(\lambda-\alpha )s}e^{\alpha s} \mathbb{E} \Vert x_{s} \Vert ^{p}_{U}\,ds \\ &\quad \leq6^{p-1}C^{p}_{g}M^{p}_{1-\beta} \lambda^{1-p\beta}\Gamma^{p-1} \biggl(\frac {p\beta-1}{p-1} \biggr)e^{(\alpha-\lambda)t} \int^{t}_{t_{1}}e^{(\lambda -\alpha)s}e^{\alpha s} \mathbb{E}\sup_{-r\leq\theta\leq0} \bigl\Vert x(s+\theta) \bigr\Vert ^{p}_{U} \,ds \\ &\qquad{}+6^{p-1}C^{p}_{g}M^{p}_{1-\beta} \lambda^{1-p\beta}\Gamma^{p-1} \biggl(\frac{p\beta-1}{p-1} \biggr) e^{(\alpha-\lambda)t} \int^{t_{1}}_{0}e^{(\lambda-\alpha)s}e^{\alpha s} \mathbb{E}\sup_{-r\leq\theta\leq0} \bigl\Vert x(s+\theta) \bigr\Vert ^{p}_{U} \,ds \\ &\quad \leq6^{p-1}C^{p}_{g}M^{p}_{1-\beta} \lambda^{1-p\beta}\Gamma^{p-1} \biggl(\frac {p\beta-1}{p-1} \biggr) e^{(\alpha-\lambda)t}e^{\alpha\theta} \int^{t}_{t_{1}}e^{(\lambda-\alpha )s}e^{\alpha(s+\theta)} \mathbb{E}\sup_{-r\leq\theta\leq0} \bigl\Vert x(s+\theta ) \bigr\Vert ^{p}_{U} \,ds \\ &\qquad{}+6^{p-1}C^{p}_{g}M^{p}_{1-\beta} \lambda^{1-p\beta}\Gamma^{p-1} \biggl(\frac{p\beta-1}{p-1} \biggr)\\ &\qquad {}\times e^{(\alpha-\lambda)t}e^{\alpha\theta} \int^{t_{1}}_{0}e^{(\lambda-\alpha )s}e^{\alpha(s+\theta)} \mathbb{E}\sup_{-r\leq\theta\leq0} \bigl\Vert x(s+\theta ) \bigr\Vert ^{p}_{U} \,ds. \end{aligned}$$

For any $x(t)\in\mathcal{S}$ and any $\varepsilon_{2}>0$, there exists $t_{2}>0$ such that $e^{\alpha s}\mathbb{E}\|x(s)\|^{p}_{U}<\varepsilon_{2}$, for $t>t_{2}$, we can get

$$\begin{aligned} &6^{p-1}C^{p}_{g}M^{p}_{1-\beta} \lambda^{1-p\beta}\Gamma ^{p-1} \biggl(\frac{p\beta-1}{p-1} \biggr) e^{(\alpha-\lambda)t}e^{-\alpha\theta}\\ &\quad {}\times\int^{t}_{t_{1}}e^{(\lambda-\alpha )s}e^{\alpha(s+\theta)} \mathbb{E}\sup_{-r\leq\theta\leq0} \bigl\Vert x(s+\theta ) \bigr\Vert ^{p}_{U} \,ds< \varepsilon_{2}. \end{aligned}$$

As $e^{(\alpha-\lambda)t}\rightarrow0$ as $t\rightarrow\infty$, there exists $t_{3}>t_{2}$ such that, for any $t\geq t_{3}$, we have

$$\begin{aligned} &6^{p-1}C^{p}_{g}M^{p}_{1-\beta} \lambda^{1-p\beta}\Gamma ^{p-1} \biggl(\frac{p\beta-1}{p-1} \biggr) e^{(\alpha-\lambda)t}e^{-\alpha\theta}\\ &\quad {}\times \int^{t_{1}}_{0}e^{(\lambda-\alpha )s}e^{\alpha(s+\theta)} \mathbb{E}\sup_{-r\leq\theta\leq0} \bigl\Vert x(s+\theta ) \bigr\Vert ^{p}_{U} \,ds< \varepsilon_{2}. \end{aligned}$$

So, from the above, we can obtain, for any $t\geq t_{3}$,

$$\begin{aligned} &6^{p-1}e^{\alpha t}\mathbb{E} \biggl\Vert \int ^{t}_{0}AS(t-s)g(s,x_{s})\,ds \biggr\Vert ^{p}_{U}\leq2\varepsilon_{2}. \end{aligned}$$

That is to say,

3.6 $$ 6^{p-1}e^{\alpha t}\mathbb{E} \biggl\Vert \int ^{t}_{0}AS(t-s)g(s,x_{s})\,ds \biggr\Vert ^{p}_{U}\longrightarrow0\quad \mbox{as }t \longrightarrow\infty. $$

Using the similar method to the forth term on the right-hand side of (3.3), we get

$$\begin{aligned} &6^{p-1} e^{\alpha t}\mathbb{E} \biggl\Vert \int ^{t}_{0}S(t-s)f(s,x_{s})\,ds \biggr\Vert ^{p}_{U} \\ &\quad \leq6^{p-1}e^{\alpha t}\mathbb{E} \biggl[ \int^{t}_{0} \bigl\Vert S(t-s)f(s,x_{s}) \bigr\Vert _{U} \,ds \biggr]^{p} \\ &\quad \leq6^{p-1}M^{p}e^{\alpha t}\mathbb{E} \biggl[ \int^{t}_{0}e^{-\lambda(t-s)} \bigl\Vert f(s,x_{s}) \bigr\Vert _{U} \,ds \biggr]^{p} \\ & \quad \leq6^{p-1}M^{p}e^{\alpha t}\mathbb{E} \biggl[ \int^{t}_{0}e^{-\lambda (p-1)(t-s)/p}e^{-\lambda(t-s)/p} \bigl\Vert f(s,x_{s}) \bigr\Vert _{U} \,ds \biggr]^{p} \\ & \quad \leq6^{p-1}M^{p}e^{\alpha t} \biggl[ \int^{t}_{0}e^{-\lambda(t-s)}\,ds \biggr]^{p-1}\mathbb{E} \biggl[ \int^{t}_{0}e^{-\lambda(t-s)} \bigl\Vert f(s,x_{s}) \bigr\Vert ^{p}_{U}\, ds \biggr] \\ &\quad \leq6^{p-1}M^{p}\lambda^{1-p}e^{\alpha t} \mathbb{E} \biggl[ \int ^{t}_{0}e^{-\lambda(t-s)} \bigl\Vert f(s,x_{s})-f(s,0)+f(s,0) \bigr\Vert ^{p}_{U} \,ds \biggr] \\ &\quad \leq12^{p-1}M^{p} C^{p}_{f} \lambda^{1-p}e^{\alpha t} \int^{t}_{-r}e^{-\lambda (t-s)}\mathbb{E} \bigl\Vert x(s) \bigr\Vert ^{p}_{U}\,ds \\ & \qquad{}+12^{p-1}M^{p}\lambda^{1-p}e^{\alpha t} \int^{t}_{0}e^{-\lambda(t-s)} \bigl\Vert f(s,0) \bigr\Vert ^{p}_{U}\,ds \\ &\quad =12^{p-1}M^{p} C^{p}_{f} \lambda^{1-p}e^{-(\lambda-\alpha)t} \int ^{t}_{-r}e^{\lambda s}e^{\alpha s-\alpha s} \mathbb{E} \bigl\Vert x(s) \bigr\Vert ^{p}_{U}\,ds \\ & \qquad{}+12^{p-1}M^{p}\lambda^{1-p}e^{-(\lambda-\alpha)t} \int ^{t}_{0}e^{\lambda s} \bigl\Vert f(s,0) \bigr\Vert ^{p}_{U}\,ds \\ &\quad \leq12^{p-1}M^{p} C^{p}_{f} \lambda^{1-p}e^{(\alpha-\lambda)t} \int ^{t}_{-r}e^{(\lambda-\alpha)s}e^{\alpha s} \mathbb{E} \bigl\Vert x(s) \bigr\Vert ^{p}_{U}\,ds \\ & \qquad{}+12^{p-1}M^{p}\lambda^{1-p}e^{-(\lambda-\alpha)t} \int ^{t}_{0}e^{\lambda s} \bigl\Vert f(s,0) \bigr\Vert ^{p}_{U}\,ds \\ &\quad :=k_{1}(t)+k_{2}(t). \end{aligned}$$

For any $x(t)\in\mathcal{S}$ and any $\varepsilon_{3}>0$, there exists $t_{4}>0$ such that $e^{\alpha s}\mathbb{E}\|x(s)\|^{p}_{U}<\frac{\lambda ^{p-1}(\lambda-\alpha)}{12^{p-1}M^{p} C^{p}_{f}}\varepsilon_{3}$, for $t>t_{4}$, we can get

$$\begin{aligned} 12^{p-1}M^{p}\lambda^{1-p}C^{p}_{f}e^{(\alpha-\lambda)t} \int ^{t}_{t_{4}}e^{(\lambda-\alpha)s}e^{\alpha s} \mathbb{E} \bigl\Vert x(s) \bigr\Vert ^{p}_{U}\, ds< \varepsilon_{3}. \end{aligned}$$

As $e^{(\alpha-\lambda)t}\longrightarrow0$ as $t\longrightarrow\infty$, there exists $t_{5}>t_{4}$ such that, for any $t\geq t_{5}$, we have

$$\begin{aligned} 12^{p-1}M^{p}\lambda^{1-p}C^{p}_{f}e^{(\alpha-\lambda)t} \int ^{t_{4}}_{-r}e^{(\lambda-\alpha)s}e^{\alpha s} \mathbb{E} \bigl\Vert x(s) \bigr\Vert ^{p}_{U}\, ds< \varepsilon_{3}. \end{aligned}$$

So, from the above, we obtain, for any $t\geq t_{5}$, $k_{1}(t) \longrightarrow0$ as $t\longrightarrow\infty$.

As $e^{-(\lambda-\alpha)t}\longrightarrow0$, as $t\longrightarrow \infty$, and condition (2.9), we can obtain $k_{2}(t)\longrightarrow0$ as $t\longrightarrow\infty$.

That is to say,

3.7 $$ 6^{p-1}e^{\alpha t}\mathbb{E} \biggl\Vert \int ^{t}_{0}S(t-s)f(s,x_{s})\,ds \biggr\Vert ^{p}_{U}\longrightarrow0\quad \text{as } t \longrightarrow\infty. $$

Using the similar method and Lemma 2.1 to the fifth term on the right-hand side of (3.3), we obtain

$$\begin{aligned} &6^{p-1} e^{\alpha t}\mathbb{E} \biggl\Vert \int ^{t}_{0}S(t-s)h(s,x_{s})\,dW(s) \biggr\Vert ^{p}_{U} \\ &\quad \leq6^{p-1}e^{\alpha t}\mathbb{E} \biggl[ \int^{t}_{0} \bigl\Vert S(t-s)h(s,x_{s}) \bigr\Vert _{U} \,dW(s) \biggr]^{p} \\ &\quad \leq6^{p-1}M^{p}e^{\alpha t}\mathbb{E} \biggl[ \int^{t}_{0}e^{-\lambda(t-s)} \bigl\Vert h(s,x_{s}) \bigr\Vert _{U} \,dW(s) \biggr]^{p} \\ &\quad \leq6^{p-1}M^{p}c_{p}e^{\alpha t} \biggl\{ \int^{t}_{0} \bigl(e^{-\lambda(t-s)}\mathbb {E} \bigl\Vert h(s,x_{s}) \bigr\Vert _{U}^{p} \bigr)^{2/p}\,ds \biggr\} ^{p/2} \\ &\quad =6^{p-1}M^{p}c_{p}e^{\alpha t} \biggl\{ \int^{t}_{0}e^{-\frac{-2\lambda (t-s)}{p}} \bigl(\mathbb{E} \bigl\Vert h(s,x_{s}) \bigr\Vert _{U}^{p} \bigr)^{2/p}\,ds \biggr\} ^{p/2} \\ &\quad \leq6^{p-1}M^{p}c_{p}e^{\alpha t} \biggl\{ \int^{t}_{0}e^{-\frac{-(2p-2)\lambda (t-s)}{p}}e^{-\frac{-2\lambda(t-s)}{p}} \bigl( \mathbb{E} \bigl\Vert h(s,x_{s}) \bigr\Vert _{U}^{p} \bigr)^{2/p}\,ds \biggr\} ^{p/2} \\ &\quad \leq6^{p-1}M^{p}c_{p}e^{\alpha t} \biggl\{ \int^{t}_{0}e^{-\frac{2(p-1)\lambda (t-s)}{p-2}}\,ds \biggr\} ^{p/2-1} \int^{t}_{0}e^{-\lambda(t-s)}\mathbb{E} \bigl\Vert h(s,x_{s}) \bigr\Vert _{U}^{p}\,ds \\ &\quad \leq6^{p-1}M^{p}c_{p}e^{\alpha t} \bigl(2 \lambda{(p-1)/(p-2)} \bigr)^{1-p/2} \int ^{t}_{0}e^{-\lambda(t-s)}\mathbb{E} \bigl\Vert h(s,x_{s})-h(s,0)+h(s,0) \bigr\Vert _{U}^{p}\,ds \\ &\quad \leq6^{p-1}M^{p}c_{p}C_{h}^{p} \bigl(2\lambda{(p-1)/(p-2)} \bigr)^{1-p/2}e^{\alpha t} \int ^{t}_{-r}e^{-\lambda(t-s)}\mathbb{E} \bigl\Vert x(s) \bigr\Vert _{U}^{p}\,ds \\ & \qquad{}+6^{p-1}M^{p}c_{p}C_{h}^{p} \bigl(2\lambda{(p-1)/(p-2)} \bigr)^{1-p/2}e^{\alpha t} \int^{t}_{0}e^{-\lambda(t-s)}\mathbb{E} \bigl\Vert h(s,0) \bigr\Vert _{U}^{p}\,ds, \end{aligned}$$

where $c_{p}=(p(p-1)/2)^{p/2}$. We remark that if $p=2$, then inequality (3.9) also holds with 0^0^:=1. Hence we have, for $p\geq2$,

3.8 $$\begin{aligned} &6^{p-1} e^{\alpha t}\mathbb{E} \biggl\Vert \int ^{t}_{0}S(t-s)h(s,x_{s})\,dW(s) \biggr\Vert ^{p}_{U} \\ &\quad \leq6^{p-1}M^{p}c_{p}C_{h}^{p} \bigl(2\lambda{(p-1)/(p-2)} \bigr)^{1-p/2}e^{\alpha t} \int ^{t}_{-r}e^{-\lambda(t-s)}\mathbb{E} \bigl\Vert x(s) \bigr\Vert _{U}^{p}\,ds \\ & \qquad{}+6^{p-1}M^{p}c_{p}C_{h}^{p} \bigl(2\lambda{(p-1)/(p-2)} \bigr)^{1-p/2}e^{\alpha t} \int^{t}_{0}e^{-\lambda(t-s)}\mathbb{E} \bigl\Vert h(s,0) \bigr\Vert _{U}^{p}\,ds. \end{aligned}$$

Similar to the proof of (3.7), from (3.8) we obtain

3.9 $$ 6^{p-1}e^{\alpha t}\mathbb{E} \biggl\Vert \int ^{t}_{0}S(t-s)h(s,x_{s})\,dW(s) \biggr\Vert ^{p}_{U}\longrightarrow0\quad \text{as } t \longrightarrow\infty. $$

As for the sixth term on the right-hand side of (3.3), by using inequality (2.2) and condition (2.7), we have

$$\begin{aligned} &\bigl(e^{\alpha t} \bigr)^{\frac{2}{p}}\mathbb{E} \biggl\Vert \int^{t}_{0}S(t-s)\sigma(s)\, dB^{H}(s) \biggr\Vert ^{2}_{U} \\ &\quad \leq \bigl(e^{\alpha t} \bigr)^{\frac{2}{p}}M^{2}c_{H}H(2H-1)t^{2H-1} \int ^{t}_{0}e^{-2\lambda(t-s)} \bigl\Vert \sigma(s) \bigr\Vert ^{2}_{L^{0}_{B}(K_{B},U)}\,ds \\ &\quad \leq M^{2}c_{H}H(2H-1)t^{2H-1}e^{({\frac{2}{p}\alpha-\lambda})t} \int ^{t}_{0}e^{\lambda s} \bigl\Vert \sigma(s) \bigr\Vert ^{2}_{L^{0}_{B}(K_{B},U)}\,ds. \end{aligned}$$

So, from the above and (2.11), we can obtain

$$\begin{aligned} \bigl(e^{\alpha t} \bigr)^{\frac{2}{p}}\mathbb{E} \biggl\Vert \int ^{t}_{0}S(t-s)\sigma(s)\,dB^{H}(s) \biggr\Vert ^{2}_{U}\longrightarrow0\quad \text{as }t \longrightarrow\infty. \end{aligned}$$

Further, by using (2.3), we get

$$\begin{aligned} &6^{p-1}e^{\alpha t}\mathbb{E} \biggl\Vert \int ^{t}_{0}S(t-s)\sigma(s)\,dB^{H}(s) \biggr\Vert ^{p}_{U} \\ &\quad \leq6^{p-1}k(p) \biggl[ \bigl(e^{\alpha t} \bigr)^{\frac{2}{p}} \mathbb{E} \biggl\Vert \int ^{t}_{0}S(t-s)\sigma(s)\,dB^{H}(s) \biggr\Vert ^{2}_{U} \biggr]^{\frac{p}{2}} \rightarrow 0\quad \text{as }t\rightarrow\infty. \end{aligned}$$

Thus, from (3.4)–(3.10), we know that $e^{\alpha t}\mathbb{E}\|(\pi x)(t)\|^{p}_{U}\longrightarrow0$ as $t\longrightarrow\infty$. So we conclude that $\pi(\mathcal{S})\subset\mathcal{S}$.

Finally, we will show that *π* is contractive. For $x,y\in \mathcal{S}$, proceeding as we did previously, we can obtain

3.10 $$\begin{aligned} &\mathbb{E} \sup_{t\in[0,T]} \bigl\Vert (\pi x) (t)-(\pi y) (t) \bigr\Vert _{U}^{p} \\ &\quad \leq4^{p-1}\mathbb{E}\sup_{t\in[0,T]} \bigl\Vert g(t,x_{t})-g(t,y_{t}) \bigr\Vert ^{p}_{U} \\ & \qquad{}+4^{p-1}\mathbb{E}\sup_{t\in[0,T]} \biggl\Vert \int ^{t}_{0}AS(t-s) (g(s,x_{s})-g(s,y_{s}) \,ds \biggr\Vert ^{p}_{U} \\ & \qquad{}+4^{p-1}\mathbb{E}\sup_{t\in[0,T]} \biggl\Vert \int ^{t}_{0}S(t-s) (f(s,x_{s})-f(s,y_{s}) \,ds \biggr\Vert ^{p}_{U} \\ & \qquad{}+4^{p-1}\mathbb{E}\sup_{t\in[0,T]} \biggl\Vert \int ^{t}_{0}S(t-s) (h(s,x_{s})-h(s,y_{s}) \,dW(s) \biggr\Vert ^{p}_{U} \\ &\quad \leq4^{p-1} \bigl\Vert (-A)^{-\beta} \bigr\Vert ^{p}_{U}C^{p}_{g} \mathbb{E}\sup _{t\in[0,T]} \bigl\Vert x(t)-y(t) \bigr\Vert _{U}^{p} \\ & \qquad{}+4^{p-1}C^{p}_{g}M^{p}_{1-\beta} \lambda^{-p\beta}\Gamma^{p-1} \biggl(\frac{p\beta-1}{p-1} \biggr) \mathbb{E}\sup_{t\in[0,T]} \bigl\Vert x(t)-y(t) \bigr\Vert _{U}^{p} \\ & \qquad{}+4^{p-1}M^{p}\lambda^{-p}C_{f}^{p} \mathbb{E}\sup_{t\in[0,T]} \bigl\Vert x(t)-y(t) \bigr\Vert _{U}^{p} \\ & \qquad{}+4^{p-1}M^{p}c_{p}C_{h}^{p} \lambda^{-p/2} \bigl(2{(p-1)/(p-2)} \bigr)^{1-p/2}\mathbb {E}\sup _{t\in[0,T]} \bigl\Vert x(t)-y(t) \bigr\Vert _{U}^{p} \\ & \quad \leq\mathbb{E}\sup_{t\in[0,T]} \bigl\Vert x(t)-y(t) \bigr\Vert _{U}^{p} \\ & \qquad{}\times4^{p-1} \biggl( \bigl\Vert (-A)^{-\beta} \bigr\Vert ^{p}_{U}C^{p}_{g}+C^{p}_{g}M^{p}_{1-\beta } \lambda^{-p\beta}\Gamma^{p-1} \biggl(\frac{p\beta-1}{p-1} \biggr) +M^{p}\lambda^{-p}C_{f}^{p} \\ & \qquad{}+M^{p}c_{p}C_{h}^{p} \lambda ^{-p/2} \bigl(2{(p-1)/(p-2)} \bigr)^{1-p/2} \biggr). \end{aligned}$$

Thus by (3.1) we know that *π* is a contraction mapping.

Hence, by the contraction mapping theorem, *π* has a unique fixed point $x(t)$ in $\mathcal{S}$, which is a solution of (2.4) with $x(s)=\varphi(s)$ on $[-r,0]$ and $e^{\alpha t}\mathbb{E}\|x(t)\| _{U}^{p}\longrightarrow0$ as $t\longrightarrow\infty$. This completes the proof. □

### Remark 3.1

Boufoussi and Hajji in [2] considered the mean square stability of NSPDE only driven by fBm. We consider the stability in the *p*th moment $(p\geq2)$ of mixed NSFPDE. In this sense, this paper generalizes the result in [2].

### Remark 3.2

When $g\equiv0, h\equiv0$ of Eq. (2.4) and $p=2$ in our paper, then inequality (3.1) can be written as $\lambda^{2}>C_{f}^{2}M^{2}$; however, the corresponding condition in Caraballo et al. [5] is $\lambda ^{2}>6c_{f}M^{2}$, where $c_{f}=C_{f}^{2}$. In addition, our condition (2.8) is

3.11 $$ \int^{t}_{0}e^{\lambda s} \bigl\Vert f(t,x_{s})-f(t,y_{s}) \bigr\Vert ^{p}_{U} \,ds\leq C^{p}_{f} \int ^{t}_{-r}e^{\lambda s} \bigl\Vert x(s)-y(s) \bigr\Vert ^{p}_{U}\,ds. $$

However, the corresponding condition in [5] is

3.12 $$ \int^{t}_{0}e^{m s} \bigl\Vert f(t,x_{s})-f(t,y_{s}) \bigr\Vert ^{2}_{U} \,ds\leq c_{f} \int^{t}_{-r}e^{m s} \bigl\Vert x(s)-y(s) \bigr\Vert ^{2}_{U}\,ds,\quad \forall 0\leq m\leq\lambda. $$

Obviously, when $p=2$, (3.11) is weaker than (3.12). So our results generalize and improve those of [5].

### Remark 3.3

When $\sigma\equiv0, p=2$, then Eq. (2.4) reduces to a NSFPDE only driven by Brownian motion in which the exponential stability in mean square of mild solution has been studied by Luo [13]. Obviously, the given result in [13] can be seen as a special case of our result. In this sense, we generalized the result given in [13].

## References

[1] Boufoussi B., Hajji S. (2011). Functional differential equations driven by a fractional Brownian motion. Comput. Math. Appl..

[2] Boufoussi B., Hajji S. (2012). Neutral stochastic functional differential equations driven by a fractional Brownian motion in a Hilbert space. Stat. Probab. Lett..

[3] Buckdahn K., Jing S. (2014). Peng’s maximum principle for a stochastic control problem driven by a fractional and a standard Brownian motion. Sci. China Math..

[4] Caraballo T., Diop M.A., Ndiaye A.A. (2014). Asymptotic behavior of neutral stochastic partial functional integro-differential equations driven by a fractional Brownian motion. J. Nonlinear Sci. Appl..

[5] Caraballo T., Garrido-Atienza M.J., Taniguchi T. (2011). The existence and exponential behavior of solutions to stochastic delay evolution equations with a fractional Brownian motion. Nonlinear Anal..

[6] Chen H., Zhu C., Zhang Y.Y. (2014). A note on exponential stability for impulsive neutral stochastic partial functional differential equations. Appl. Math. Comput..

[7] Cheridito P. (2001). Mixed fractional Brownian motion. Bernoulli.

[8] Da Prato G., Zabczyk J. (1992). Stochastic Equations in Infinite Dimensions.

[9] Govindan T.E. (2005). Almost sure exponential stability for stochastic neutral partial functional differential equations. Stochastics.

[10] Guerra J., Nualart D. (2008). Stochastic differential equations driven by fractional Brownian motion and standard Brownian motion. Stoch. Anal. Appl..

[11] Li Z. (2015). Global attractiveness and quasi-invariant sets of impulsive neutral stochastic functional differential equations driven by fBm. Neurocomputing.

[12] Liu K., Xia X. (1999). On the exponential stability in mean square of neutral stochastic functional differential equations. Syst. Control Lett..

[13] Luo J. (2009). Exponential stability for stochastic neutral partial functional differential equations. J. Math. Anal. Appl..

[14] Luo J., Taniguchi T. (2009). Fixed points and stability of stochastic neutral partial differential equations with infinite delays. Stoch. Anal. Appl..

[15] Mémin J., Mishura Y., Valkeila E. (2001). Inequalities for the moments of Wiener integrals with respect to a fractional Brownian motion. Stat. Probab. Lett..

[16] Mishura Y., Shevchenko G. (2011). Mixed stochastic differential equations with long-range dependence: existence, uniqueness and convergence of solutions. Comput. Math. Appl..

[17] Mishura Y.S., Posashkova S.V. (2011). Stochastic differential equations driven by a Wiener process and fractional Brownian motion: convergence in Besov space with respect to a parameter. Comput. Math. Appl..

[18] Pazy A. (1983). Semigroups of Linear Operator and Applications to Partial Differential Equations.

[19] Randjelović J., Janković S. (2007). On the pth moment exponential stability criteria of neutral stochastic functional differential equations. J. Math. Anal. Appl..

[20] Shevchenko G. (2014). Mixed fractional stochastic differential equations with jumps. Stochastics.

[21] Shevchenko G., Shalaiko T. (2013). Malliavin regularity of solutions to mixed stochastic differential equations. Stat. Probab. Lett..

